# Identification and selection of normalization controls for quantitative transcript analysis in *B* *lumeria graminis*

**DOI:** 10.1111/mpp.12300

**Published:** 2015-10-09

**Authors:** Helen G. Pennington, Linhan Li, Pietro D. Spanu

**Affiliations:** ^1^ Department of Life Sciences Imperial College London London SW7 2AZ UK

**Keywords:** actin, glyceraldehyde‐3‐phosphate dehydrogenase, histone 3, normalization, qPCR, tubulin, ubiquitin

## Abstract

The investigation of obligate biotrophic pathogens, for example *B* *lumeria graminis*, presents a number of challenges. The sensitivity of many assays is reduced because of the presence of host material. Furthermore, the fungal structures inside and outside of the plant possess very different characteristics. Normalization genes are used in quantitative real‐time polymerase chain reaction (qPCR) to compensate for changes as a result of the quantity and quality of template material. Such genes are used as references against which genes of interest are compared, enabling true quantification. Here, we identified six potential *B* *. graminis* and five barley genes for qPCR normalization. The relative changes in abundance of the transcripts were assayed across an infection time course in barley epidermis, in *B* *. graminis* epiphytic structures and haustoria. The *B* *. graminis* glyceraldehyde‐3‐phosphate dehydrogenase (GAPDH), actin (ACT) and histone 3 (H3) genes and the barley GAPDH, ubiquitin (UBI) and α‐tubulin 2B (TUBA2B) genes were optimal normalization controls for qPCR during the infection cycle. These genes were then used for normalization in the quantification of the members of a Candidate Secreted Effector Protein (CSEP) family 21, a conidia‐specific gene and barley genes encoding putative interactors of CSEP0064. The analysis demonstrates the importance of identifying which reference genes are appropriate for each investigation.

## Introduction

Powdery mildew of grasses and cereals is caused by the obligate biotrophic fungus *Blumeria graminis* (DC) Speer. This fungus possesses high host specificity: different host genera are infected by each of eight *formae speciales* (f. sp.) (Troch *et al*., 2014). *Blumeria graminis* f. sp. *hordei*, referred to here as *B. graminis*, infects barley and has a great economic impact, causing substantial crop losses every year. It can be considered as a model organism for the powdery mildews (Bindschedler *et al*., 2009; Both and Spanu, 2004), and was the first powdery mildew to be sequenced and annotated (Spanu and Kaemper, 2010; Spanu *et al*., 2010).

Like many fungal pathogens, *B. graminis* is an obligate biotroph: it cannot be cultured outside its host (Spanu and Kaemper, 2010). The fungal structures present inside and outside the host possess very different characteristics. For *B. graminis*, external features include spores, primary and secondary germ tubes, and surface hyphae, whereas haustoria are formed inside barley epidermal cells (Both and Spanu, 2004). During infection with *B. graminis,* significant changes in transcript abundance take place (Both *et al*., 2005). The prerequisite alterations in gene activity that cause these changes mean that the abundance of housekeeping gene transcripts for host and pathogen cannot be simply assumed to be expressed at constant levels throughout.

In common with other haustorium‐forming pathogens (O'Connell and Panstruga, 2006; Panstruga and Dodds, 2009), *B. graminis* and its host are engaged in ‘secretory warfare’, with small effector proteins being delivered at the haustorial complex. There are 491 Candidate Secreted Effector Proteins (CSEPs) in *B. graminis* which have no blast hits outside of the mildews (Ersiphales), do not have transmembrane domains and possess a predicted signal peptide (Panstruga and Dodds, 2009; Spanu and Kaemper, 2010; Spanu *et al*., 2010). The silencing of eight *Blumeria* Effector Candidates (BECs) (BEC1005, BEC1016, BEC1018, BEC1019, BEC1038, BEC1040, BEC1054 and its paralogue BEC1011) by host‐induced gene silencing (HIGS) determines a reduction in the haustorial index (HI) (Pliego *et al*., 2013). The BEC and CSEP sets overlap, containing many of the same proteins, and they both demonstrate great sequence diversity. One such family is CSEP family 21, which includes the RNAse‐like proteins CSEP0064 (BEC1054), CSEP0065, CSEP0066 and CSEP0264 (BEC1011; which has the same nucleotide sequence as CSEP0486). Here, we quantified the levels of RNAs encoding members of this CSEP family, together with a conidia‐specific gene. A number of host proteins that may interact with CSEP0064 have been identified (H. G. Pennington *et al*., unpublished data). The levels of mRNA corresponding to these barley genes were also measured.

Quantitative real‐time PCR (qPCR) allows mRNA transcripts to be quantified simultaneously for a number of genes across many different samples (Fink *et al*., 1998; Heid *et al*., 1996; Higuchi *et al*., 1993). Furthermore, it can be considered to be both quick and amenable to relatively high throughput compared with conventional methods for RNA quantification, such as ribonuclease protection assays, northern blots or competitive reverse transcription‐polymerase chain reaction (RT‐PCR) (Vandesompele *et al*., 2002). When determining changes in transcript abundance through qPCR, a number of variables need to be monitored, including template quantity and quality (Vandesompele *et al*., 2002). Traditional methods for the normalization of transcript abundance include the determination of cell number (difficult for solid samples, such as barley leaves), RNA mass quantity (which does not assess the quality or enzymatic efficiency) and the amount of 18S/28S RNA (where the total amounts can never be removed, only reduced) (Vandesompele *et al*., 2002). For qPCR, normalization is usually carried out through the use of internal control genes, often referred to as ‘housekeeping’ genes. Such genes are used as references against which genes of interest can be compared. Ideally, they should be stably expressed in the tissues under investigation, regardless of the experimental treatment. Validation of the stability of these genes should be performed as their expression can vary considerably (Bustin, 2000; Suzuki *et al*., 2000; Thellin *et al*., 1999; Warrington *et al*., 2000).

Vandesompele *et al*. (2002) addressed the question of how to validate the expression of a control gene without the availability of a reliable measure with which to normalize the control. Their method relies on the principle that the expression ratios of two ideal control genes would be identical, irrespective of cell type or experimental conditions. Variation in the expression ratios of housekeeping genes indicates that one or more of the housekeeping genes are not constantly expressed, with an increasing ratio corresponding to decreasing expression stability.

In this study, we identified six candidate control genes for *B. graminis* qPCR and five for barley based on those used in published papers. The stability of these genes was then experimentally determined for both epidermal and epiphytic tissues during infection by *B. graminis*. Our aim was to identify transcripts whose abundance varied as little as possible, and which were most suited for the normalization of other *B. graminis* and barley genes.

## Results

### Selection of control genes

The genes identified/selected for use with *B. graminis* were α‐tubulin (TUBA; Hacquard *et al*., 2013), β‐tubulin (TUBB; Hacquard *et al*., 2013; Zhang *et al*., 2000), actin (ACTB; Hacquard *et al*., 2013), glyceraldehyde‐3‐phosphate dehydrogenase (GAPDH; Hacquard *et al*., 2013), histone 3 (H3; Both *et al*., 2005) and monoglyceride lipase (MGLL; Both *et al*., 2005; Nowara *et al*., 2010). In addition, TUBB, H3 and MGLL were previously identified as stable genes during the *B. graminis*–barley interaction (Both *et al*., 2005). The three genes TUBA, TUBB and ACTB have all been used in other fungal species, including *Melampsora larici‐populina* (Hacquard *et al*., 2011), *Fusarium graminearum* (Brown *et al*., 2011), *Magnaporthe oryzae* (Kim *et al*., 2009), *Cladosporium cladosporioides*, *Aspergillus niger* and *Penicillium chrysogenum* (Ettenauer *et al*., 2014).

The five barley control genes used were ubiquitin (UBQ; Besse *et al*., 2011; Trujillo *et al*., 2006), adenosine triphosphatase (H^+^‐ATPase; Besse *et al*., 2011), GAPDH Besse *et al*., 2011; Ma *et al*., 2013; a related GAPDH, Accession AK251456 (identities 813/987 (82%), query cover 71%, blastn‐2‐sequences), was used elsewhere (Jarosova and Kundu, 2010)], ACTB (Jiang *et al*., 2011; Ma *et al*., 2013) and TUBA (Besse *et al*., 2011; Doblin *et al*., 2009). A related TUBA, Accession AK260165 (identities 1600/1602 (99%), query cover 98%, blastn‐2‐sequences), was also tested as a control.

**Figure 1:**
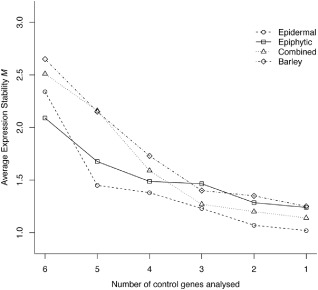
Calculation of control gene stability (*M*). The average internal control gene stability was calculated for quantitative real‐time polymerase chain reaction (qPCR) control genes as the pairwise variation of a control gene with all other control genes. The least stable control was then excluded on the basis of the average pairwise variation. The least stable genes have the highest *M* value and the most stable genes have the lowest *M* value. The calculation and exclusion process was repeated in a stepwise manner, resulting in two control genes remaining. The *x* axis represents the ranking of the control genes in order of increasing stability from left to right. Six control genes were analysed for each of the following materials: *B* *lumeria graminis* epidermal material (Epidermal), *B* *. graminis* epiphytic material (Epiphytic), the combined *B* *. graminis* epidermal and epiphytic material (Combined) and barley epidermal material (Barley).

The average internal control gene stability (*M*) was calculated as the pairwise variation of a control gene with all other control genes. The *M* value was derived from the standard deviation of the logarithmically transformed ratios of the transcript quantities. A low *M* value indicates stable expression and a high *M* value indicates variation in transcript abundance. The *M* value was calculated, the least stable gene was excluded on the basis of the average pairwise variation and the process was repeated until the two most stable genes with the lowest *M* value were obtained (Table 1 and Fig. 1). Stepwise exclusion of controls with the largest *M* value eventually leads to the selection of the two control genes that are most stably expressed. The algorithm used was originally unable to rank the last two genes (Table 1), as exclusion is calculated on the basis of the average pairwise variation (Vandesompele *et al*., 2002). This problem has since been rectified through improvement of the gene normalization (geNorm) software, allowing the identification of the most stable gene (qbase+ v3.0, Biogazelle, http://www.qbaseplus.com).

**Table 1:** Barley and *B* *lumeria graminis* control genes ranked in order of expression stability. The genes are listed from 1–6, with ‘1’ being the most stable and ‘6’ being the least stable

Rank	*Blumeria graminis* reference genes			Barley reference genes
	Epidermal	Epiphytic	Combined	Epidermal
1	ACT	MGLL	GAPDH	GAPDH
2	GAPDH	TUBA	ACT	UBI
3	H3	GAPDH	H3	TUBA2B
4	MGLL	ACT	MGLL	TUBA
5	TUB2B	H3	TUB2B	ATPase
6	TUBA	TUB2B	TUBA	ACT

ACT, actin; GAPDH, glyceraldehyde‐3‐phosphate dehydrogenase; H3, histone 3; MGLL, monoglyceride lipase; TUB, tubulin; UBI, ubiquitin.

In the conditions used here, the control genes ACT, GAPDH and H3 were the most stable in both *B. graminis*‐infected epidermis samples and in the ‘combined analysis’ sample (where *B. graminis* epidermal and epiphytic material was analysed together; Table 1). In contrast, in epiphytic *B. graminis*, levels of TUBA, MGLL and GAPDH transcripts varied least. In infected barley epidermis, levels of GAPDH, UBI and TUBA2B transcripts were found to be the least variable.

### Number of control genes

**Figure 2:**
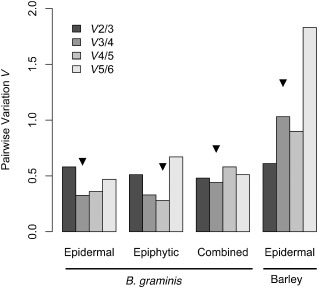
Determination of how many genes should be used for gene normalization. The number of control genes required for accurate normalization was determined through pairwise variation analysis, where high variation values (*V*) correspond to low correlation coefficients. The term ‘epidermal’ refers to *B* *lumeria graminis* present in barley epidermal tissue, ‘epiphytic’ to *B* *. graminis* present on the surface of the leaf, ‘combined’ to the analysis of the epidermal and epiphytic material together, and ‘barley’ to barley epidermal material. The symbol ‘▼’ indicates the optimal number of control genes for normalization. The optimal number of reference genes for normalization was calculated using the pairwise variation (*V* *_n_*_/_ *_n_*_+ 1_) between the normalization factors NF and NF*_n_*_+ 1_ (where *n* is the number of genes) (for details, see Vandesompele *et al*., 2002).

The optimal number of reference genes for normalization was calculated using the ‘pairwise variation’, defined as *V*_*n*/*n* + 1_ (where *n* is the number of genes and 3 ≤ *n* ≤ 5; Vandesompele *et al*., 2002). A large variation in *V* indicates that the added control gene has a significant effect. A plot of the *V* values in the various tissue samples (Fig. 2) indicates that the optimal numbers of control genes for *B. graminis* epidermal, epiphytic and combined qPCR substrates are three, four and three, respectively. Furthermore, three control genes were found to be the optimal number for barley epidermis.

Vandesompele *et al*. (2002) recommended the use of a minimum of three control genes for normalization. We used the best three control genes to normalize the expression of *B. graminis* and barley genes.

### Control gene expression

**Figure 3:**
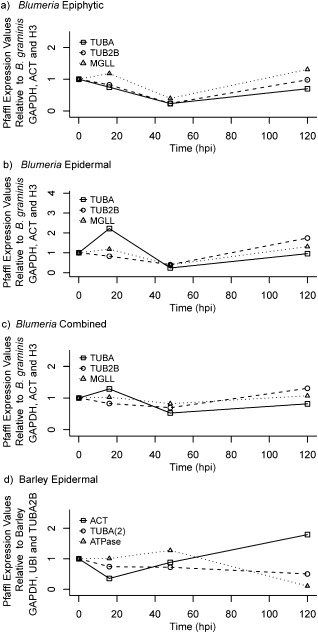
Quantification of RNA/cDNA corresponding to candidate ‘control’ genes. The RNA levels, shown as Pfaffl expression values, are relative to 0 h post‐inoculation (hpi) (=1) for each of the controls in tissues sampled from 0 to 120 hpi. The values correspond to control genes of *B* *lumeris graminis* from epiphytic material only (a), infected epidermis only (b), combined epidermal and epiphytic material (c) and barley control genes in infected epidermis (d). ACT, actin; GAPDH, glyceraldehyde‐3‐phosphate dehydrogenase; H3, histone 3; MGLL, monoglyceride lipase; TUB, tubulin; UBI, ubiquitin.

The Pfaffl values for the remaining control genes were calculated and plotted in relation to the three optimal controls (Fig. 3). In epiphytic material (Fig. 3a), expression of the control gene MGLL increased at 16 h post‐inoculation (hpi) and then decreased by 48 hpi without returning to pre‐infection levels of expression. The transcript abundance decreased for both TUBA and TUB2B, with a minimum expression level at 48 hpi before increasing again by 120 hpi. This expression pattern for TUB2B was found to be the same in both epidermal and combined material (Fig. 3b,c). In both combined and epidermal material, the transcript abundance of TUBA increased at 16 hpi before decreasing (Fig. 3b,c), and then continued to remain below its original expression level. The levels of MGLL transcript also varied, showing an initial increase at 16 hpi, a decrease at 48 hpi and a further increase at 120 hpi.

For barley (Fig. 3d), expression of TUBA decreased by 16 hpi, and then remained relatively constant; ACT decreased by 16 hpi, and then increased at both 48 hpi and 120 hpi. The expression of ATPase increased at 16 hpi and 48 hpi, and then decreased by 120 hpi.

### CSEP expression

**Figure 4:**
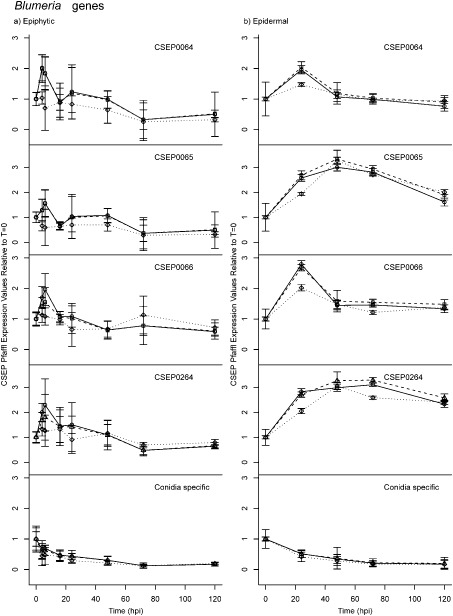
Expression of *B* *lumeria graminis* Candidate Secreted Effector Proteins (CSEPs) from effector family 21 during infection. The levels of the various effectors, shown as Pfaffl expression values, were determined through quantitative real‐time polymerase chain reaction (qPCR) for *B* *. graminis* from epiphytic material (a) or from infected epidermis only (b). The RNA levels are relative to 0 h post‐inoculation (hpi) (=1) for each of the controls in tissues sampled from 0 to 120 hpi. The control genes for normalization were glyceraldehyde‐3‐phosphate dehydrogenase (accession CCU80715), actin (accession CCU76638) and histone‐3 (accession CCU82905). Error bars represent the standard deviation of three biological replicates.

We measured the RNA abundance of CSEP family 21 and of the conidia‐specific gene during the initial stages of powdery mildew development (Fig. 4). All four CSEPs showed the same general trend in epiphytic material: a maximum expression at 16 h, followed by a broad secondary peak in expression at 24 hpi (CSEP0066) or from 24 to 48 hpi (CSEP0064, CSEP0065 and CSEC0246). In epidermal material, CSEP0064 and CSEP0066 both showed maximum expression at 48 hpi, followed by a decrease in expression to near the original expression level. In contrast, CSEP0065 and CSEP0264 both increased in expression, with the highest level at 48 hpi, and then decreased in expression by 120 hpi. For all of the CSPEs, the use of the two best controls (ACT and GAPDH), instead of three (ACT, GAPDH and H3), produced the same general trends. The use of the worst control (TUB2B) produced a different series of results: the second peak was lost/obscured for all CSEP epiphytic material. This change was the most dramatic for CSEP0066 and CSEP0264. CSEP0066 gained an additional secondary peak.

The conidia‐specific gene was found to decrease in abundance in both epidermal and epiphytic material following infection, when normalized against the best (ACT, GAPDH and H3) or worst (TUB2B) control genes.

**Figure 5:**
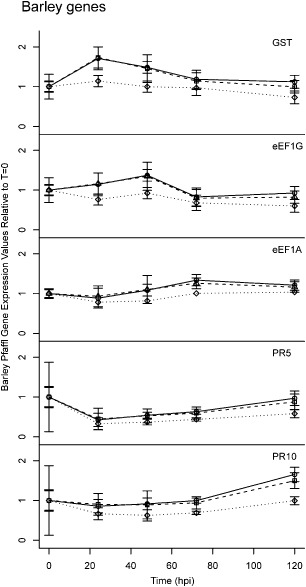
Expression of barley genes during infection. The levels of the various genes, shown as Pfaffl expression values, were determined through quantitative real‐time polymerase chain reaction (qPCR) for barley from epidermal material. The RNA levels are relative to 0 h post‐inoculation (hpi) (=1) for each of the controls in tissues sampled from 0 to 120 hpi. The control genes for normalization were glyceraldehyde‐3‐phosphate dehydrogenase (accession X60343), ubiquitin (accession X04133) and tubulin A (accession U40042). Error bars represent the standard deviation of three biological replicates. GST, glutathione‐S‐transferase; eEF1G eukaryotic elongation factor 1 gamma; eEF1A, eukaryotic elongation factor 1 alpha; PR, pathogenesis related protein.

The transcript abundance of selected barley genes is shown in Fig. 5. Glutathione‐S‐transferase (GST), eukaryotic elongation factor 1 gamma (eEF1G) and eukaryotic elongation factor 1 alpha (eEF1A) RNA increased by 24, 48 and 72 hpi, respectively (Fig. 5), and then decreased to near the original expression levels. In contrast, transcript abundance for pathogenesis related protein (PR) 5 and PR10 decreased initially and then increased, reaching the highest levels at 120 hpi. In PR10, only the final levels at 120 hpi were higher than the initial level. The use of the worst control was found, for all genes investigated, to obscure most of the change in transcript abundance.

## Discussion

Accurate normalization controls are essential for the reliable quantification of gene expression, especially when attempting to determine subtle changes. This issue has been increasingly highlighted in the literature; numerous studies have indicated that housekeeping transcript abundance can vary considerably (reviewed in Vandesompele *et al*., 2002).

The use of multiple control genes for qPCR normalization is recommended: together, this reduces the error associated with the use of single controls (Vandesompele *et al*., 2002). Six potential normalization genes from *B. graminis* and five from barley were investigated in both epiphytic and infected epidermis during a time course of infection. Following the selection of optimal normalization controls (Table 1), the Pfaffl values for the RNA of the remaining control genes were calculated, and plotted in relation to the optimal controls (Fig. 3). Our results indicated that the abundance of the transcripts varied between control genes.

An increase in TUBA accumulation is seen in epidermal and combined material when compared with the optimal controls GAPDH, ACT and H3 at 16 hpi. This may reflect the cytoskeletal changes which take place as the fungus penetrates the host cell and forms complex multidigitated fungal haustoria (Spanu and Kaemper, 2010).

Obligate biotrophs present a number of challenges: they cannot be cultured outside their host; their life cycle is closely tied to the infection process and the host's response; and the structures inside and outside the host differ (Spanu and Kaemper, 2010). As *B. graminis* grows only within epidermal cells, dissection of the epidermis reduces the noise caused by the presence of host tissue, but the fungus still represents only a small percentage of the total biomass. The variation between tissue sources and cell type is exemplified by different normalization optima found for the different tissues (Fig. 3 and Table 1). The control genes GAPDH, ACT and H3 were used for normalization of transcript abundance, as they were the most stable for both epiphytic material and for the combined epiphytic and epidermal samples (Table 1).

In contrast with the results presented here, in a previous qPCR study, the *B. graminis* ACT and TUBB genes were the two most stable controls (compared with TUBA, elongation factor 1α, ubiquitin conjugating enzyme E2 and GAPDH; Hacquard *et al*., 2013). We found TUB2B to be the *least* stable control gene in epidermal material. These differences may have been caused by the sample materials (whole leaf as opposed to separate epidermal/epiphytic tissues), control genes or time scales used. The use of the least stable control genes was found to obscure changes in the expression levels of some genes and, in the case of CSEP0066 and CSEP0264, to produce secondary peaks which could not be identified when the results were normalized against the other controls (Figs 4 and 5). This highlights the need to identify control genes appropriate to the experiment, and ultimately to the question under investigation.

For barley, GAPDH, UBI and TUBA2B were the three most stable RNAs during infection, and ACT was the *least* stable of the control RNAs (in contrast with *B. graminis*, where ACT was one of the two *most* stable RNAs). The barley controls also demonstrated a different pattern of expression (Fig. 3). The increase observed in transcript levels of the barley control genes ACT and ATPase at 48 hpi correlates with haustorial formation, and therefore with the timing of the formation of the extrahaustorial membrane (O'Connell and Panstruga, 2006; Panstruga and Dodds, 2009). Epidermal cells respond to attempted pathogen penetration through cytoskeletal rearrangement (Gross *et al*., 1993; Kobayashi *et al*., 1992). The up‐regulation of cytoskeletal genes may reflect these changes taking place inside the host cells during infection (Both *et al*., 2005).

A recent study (Ferdous *et al*., 2015) has also investigated the use of the normalization control genes ACT, TUBA and GAPDH, together with small nucleolar RNAs and microRNAs, under a range of stress treatments, including infection with the necrotrophic fungal pathogen *Rhynchosporium commune*. They found that ACT was more stable than GAPDH, which, in turn, was more stable than TUBA. In contrast, we found that TUBA was the most stable of the three, and ACT the least stable. This difference in results may be caused by the use of a different fungal pathogen or different treatment conditions. Furthermore, their results showed that the order of stability varied under different stress treatments, with differing control genes performing best under different conditions, although they did highlight the potential for use of small nucleolar RNAs and an ADP‐ribosylation factor‐1‐like protein in qPCR normalization.

The four CSEPs investigated in this study showed similar expression patterns in epiphytic material, with expression peaks at 16 hpi and either 24 or 48 hpi. These two time points represent penetration peg formation and the stage between infection and colonies becoming visible to the naked eye on the leaf's surface (Both *et al*., 2005). The presence of the early peaks indicates that these CSEPs may play a role in more than one stage during the infection process. In general, our results for CSEP0064 and CSEP0066 support those published previously (Pliego *et al*., 2013), with the second peak occurring at the same time (*c*. 24 hpi) as the peak detected by Pliego *et al*. (2013). Similarly, both CSEP0064 and CSEP0066 showed a maximum peak in transcript abundance in epidermal material at 24 hpi. The expression patterns in epidermal material for CSEP0065 and CSEP0264 were different from those in epiphytic material, with generally elevated levels of each CSEP, and a peak in abundance at 48 hpi. These results suggest that these CSEPs play a role in the later stages of infection.

In contrast with the CSEP results, the conidia‐specific gene did not show a peak in transcript abundance. This indicates that the peak of transcript accumulation detected at 16 hpi in both epidermal and epiphytic samples is biologically relevant and not caused by a normalization bias.

The five barley genes have been identified in a previous study as possible interactors for CSEP0064 (BEC1054) (H. G. Pennington *et al*., unpublished data). Their expression patterns during infection varied considerably (Fig. 5). The increase in transcription for GST, eEF1G and eEF1A corresponds to the peaks in abundance for CSEP0064 in both epiphytic and epidermal material. In contrast, PR5 and PR10 were reduced at almost all time points compared with uninfected samples at the start of the infection.

## Experimental Procedures

Chemicals were obtained from VWR (Lutterworth, Leicestershire, UK) and materials/reagents for qPCR were obtained from PrimerDesign (Southampton, Hampshire, UK) unless stated otherwise.

### Plant and fungal materials

Barley (*Hordeum vulgare* L. cv. Golden Promise) was cultivated in 13‐cm‐diameter pots filled with Levingtons F2+S compost. Seedlings were transferred 7 days post‐germination to 60‐cm^3^ Perspex boxes, and inoculated with *Blumeria graminis* f. sp. *hordei* DH14. Plants were grown under a long‐day cycle (16 h light, 8 h darkness) with 33% humidity at 25 °C.

### Sample collection

Conidia were collected with a vacuum pump. All other material was collected by immersing barley leaves in 5% cellulose acetate dissolved in anhydrous acetone, leaving the acetone to evaporate and then collecting the cellulose acetate (which contained the epiphytic material). Following this, epidermal peels were performed to obtain barley epidermal material (which contains *B. graminis* haustoria). Samples were flash frozen in liquid nitrogen and stored at −80 °C until further use.

### Selected time points

Samples were collected for the geNorm experiments from ungerminated conidia (i.e. 0 hpi), at 16 hpi (penetration peg formation), at 2 days post‐inoculation (dpi; new colony formation) and at 5 dpi (colonies become abundant on the leaf surface), as these time points represent the beginning, middle and end of the asexual infection cycle. In addition to the times used for the geNorm assay, the activity of members of CSEP family 21 was measured at 4 hpi (germinated conidia with primary and secondary appressorial germ tubes), 6 hpi (appressorium formation), 24 hpi (haustorial formation) and 3 dpi (colonies visible to the naked eye) (Both and Spanu, 2004). For the geNorm assay, two biological replicates were used for each time point, as recommended by PrimerDesign.

### Extraction and analysis of RNA

Samples (either cellulose acetate containing epiphytic material or epidermal peels) were ground in liquid nitrogen with a mortar and pestle following the addition of quartz sand (50–70 mesh; Sigma‐Aldrich, St Louis, MO, USA; catalogue number 274739) and then extracted using the QIAGEN RNEasy Mini Kit (QIAGEN, Crawley, UK; catalogue number 74104) according to the manufacturer's instructions, but with the following changes: the ground material was incubated in buffer RLT (QIAGEN) for 20 min and centrifuged at maximum speed for 20 min before being transferred to the QIA shredder spin column. In addition, the material was washed twice with buffer RW1 (QIAGEN). These modifications were found to provide a higher yield of RNA.

### RNA quality control

The quantity of the RNA was determined using a NanoDrop‐1000 spectrophotometer (Thermo Scientific, Wilmington, ME, USA) immediately post‐extraction, and RNAs with an optical density at 260/280 nm (OD_260/280_) of greater than 1.8 and an OD_260/230_ of greater than 1.5 were used for further work (Manchester, 1996; Sambrook and Russell, 2001). Samples were then analysed using an Agilent RNA 6000 Nano Kit (Agilent Technologies, Santa Clara, CA, USA; catalogue number 5067‐1511), with an Agilent 2100 Bioanalyzer. The RNA Integrity Number (RIN) is a widely used criterion for the assessment of RNA integrity, which is scored from 1–10, with 10 representing the most intact RNA. For this study, only RNA with RIN > 6.5 was used for further investigation (Fleige and Pfaffl, 2006) [see Table S5 (Supporting Information) for the yield and RIN numbers of all samples used].

### Reverse transcription

The RNA was treated with DNase (Precision DNase Kit, PrimerDesign, catalogue number DNASE50) to remove genomic DNA. Complementary DNA (cDNA) was then synthesized using the Precision nanoScript 2 Reverse Transcription Kit (PrimerDesign, catalogue number RT‐nanoscript2) with a 1 : 1 mixture of random nonamer and Oligo‐dT primers, according to the manufacturer's instructions. The cDNA samples were then stored at −20 °C until further use. The primers used for qPCR are given in Tables S1–S3 (see Supporting Information).

qPCR was performed in a 7500‐Fast Thermocycler (ThermoScientific, Loughborough, UK) using a PrecisionFAST Mastermix SYBR green detection kit (PrimerDesign, catalogue number PrecisionFAST), with 25 ng of cDNA template and 1 μL of primer/probe mix in a 20‐μL reaction manually added to BrightWhite Real‐Time PCR plates (PrimerDesign, catalogue number BW‐FAST), according to the manufacturer's instructions. The following conditions were used: 95 °C for 2 min, followed by 40 cycles of 95 °C for 5 s and 59 °C for 60 s. For the qPCR experiments using CSEP primers (Table S3) and barley genes (Table S4), the conditions were the same, but with a working concentration of 300 nm of primers and 3 pmol of probe, and the CSEP primers were ordered, with desalting purification, from Invitrogen (Invitrogen, Carlsbad, CA, USA). PrimerDesign performed validation of the control primers. The primers for CSEPs were analysed through gradient PCR to identify the optimal annealing temperatures, and an average temperature of 59 °C was used. The primers were found to give a single clean band of the expected size corresponding to the predicted amplicon length. Furthermore, all primer pairs were found to give a single melt curve peak with no shoulder.

Three biological replicates were used for each time point for the CSEPs, and biological samples were treated as independent samples in further analysis. Calculations of *C*_T_ values and initial analyses were performed using 7500‐Fast Software v1.0 (ThermoScientific). No amplification was found for the ‘no‐template’ controls for either the control primers or the CSEPs. Analyses of the controls were performed using geNorm software (Vandesompele *et al*., 2002). Additional comparisons were performed between the controls, and between the CSEPs and the controls, using the Pfaffl method, on the averages of the biological replicates, where (Livak and Schmittgen, 2001):
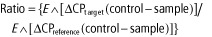



## Conclusion

Six housekeeping genes from *B. graminis* and five from barley were assayed to determine their suitability as normalization controls for qPCR during an infection time course. The best housekeeping genes identified from this investigation were ACT, GAPDH and H3, and the best genes for barley were GAPDH, UBI and TUBA2B. Our results demonstrate that housekeeping genes can vary widely between tissues, and between species, highlighting the need to identify controls appropriate to each investigation.

## Supporting information


**Table S1** *Blumeria graminis* housekeeping gene primers.
**Table S2** *Hordeum vulgare* housekeeping gene primers.
**Table S3** Primers used for *Blumeria graminis* Candidate Secreted Effector Protein (CSEP) family 21 and conidia‐specific gene quantitative real‐time polymerase chain reaction (qPCR).
**Table S4** Primers used for *Hordeum vulgare* gene quantitative real‐time polymerase chain reaction (qPCR).
**Table S5** Analyses of RNA samples used for quantitative real‐time polymerase chain reaction (qPCR). RIN, RNA integrity number; hpi, hours post‐inoculation; epiphytic, *Blumeria graminis* epiphytic material; ‘epidermal’, barley epidermal peels (containing *B. graminis* hyphae).
