# Supplementary material for: Identification and selection of normalization controls for quantitative transcript analysis in B lumeria graminis
Source: Mol Plant Pathol. 2015 Oct 9;17(4):625–33. doi: 10.1111/mpp.12300 (PMC5102671; doi:10.1111/mpp.12300)
Supplement: Supplementary file 1 — Table S1 Blumeria graminis housekeeping gene primers. Table S2 Hordeum vulgare housekeeping gene primers. Table S3 Primers used for Blumeria graminis Candidate Secreted Effector Protein (CSEP) family 21 and conidia‐specific gene quantitative real‐time polymerase chain reaction (qPCR). Table S4 Primers used for Hordeum vulgare gene quantitative real‐time polymerase chain reaction (qPCR). Table S5 Analyses of RNA samples used for quantitative real‐time polymerase chain reaction (qPCR). RIN, RNA integrity number; hpi, hours post‐inoculation; epiphytic, Blumeria graminis epiphytic material; ‘epidermal’, barley epidermal peels (containing B. graminis hyphae). [file MPP-17-625-s001.docx]

# Supporting Information

**Supporting Table 1**: *B. graminis* housekeeping gene primers.

| Accession (protein) | Accession (cDNA) | gene | abbreviation | FWD Sequence (5’-3’) | REV sequence (5’-3’) | Amplicon length | Efficiency |
| --- | --- | --- | --- | --- | --- | --- | --- |
| CCU82444 | CAUH01006388 | α-tubulin | TUBA | GGTCACTACACTGTTGGTAAAGA | CCGAAGGAATGGAATACAAGAAAG | 110 | 1.23 |
| CAA35709 | X51326 | β-tubulin | TUBB | GGAAACGCACCTACACTATACC | GCCGTGATGGAATTTAACTAACAA | 94 | 1.19 |
| CCU76638 | CAUH01002841 | actin | ACTB | CCCAATTTACGAAGGTTTCTCTC | TCAGCGGTTGTGGAAAAAGT | 126 | 1.21 |
| CCU80715 | CAUH01004767 | glyceraldehyde 3-phosphate dehydrogenase | GAPDH | GGAGCCGAGTACATAGTAGAGT | GGAGGGTGCCGAAATGATAAC | 105 | 1.23 |
| CCU82905 | CAUH01007168 | Histone 3 | H3 | GGAAACAACTCGCTTCTAAGG | GATTTTTGGTATCTTCTGATTTCAC | 121 | 1.22 |
| CCU80195 | CAUH01004592 | monoglyceride lipase | MGLL | GCCCTACCAGCCGAAAAC | ATGCCTGATAATCCCTCTAACG | 104 | 1.24 |

**Supporting Table 2**: *H. vulgare* housekeeping gene primers.

| Accession | gene | abbreviation | FWD Sequence (5’-3’) | REV sequence (5’-3’) | Amplicon length | Efficiency |
| --- | --- | --- | --- | --- | --- | --- |
| U40042 | a-tubulin | TUBA | CAACATACACCAACCTCAACAG | AACTCATTCACATCAACATTCAGA | 100 | 1.23 |
| AY145451 | actin | ACTB | CTGTGCCCATTTATGAAGGATAC | GCTGAGGTTGTGAAGGAGTAA | 127 | 1.22 |
| X04133 | ubiquitin | UBQ | TTTGGTATTATTGAGGGTCTGATGA | TGCTGCTGGGGATGATGTT | 130 | 1.25 |
| AJ344078 | adenosine triphosphatase | H+-ATPase | TCTCAGGGTTCACAGGTCTT | CCGAACAGGTCCGTAATGG | 93 | 1.24 |
| X60343 | glycerinaldehyde-phosphate dehydrogenase | GAPDH | CTGATTGAGAAGGCTGATGGAT | AGAGCAGGAGCGTCATTGA | 128 | 1.25 |

**Supporting Table 3** Primers used for *B. graminis* CSEP family 21 and conidia-specific gene qPCR.

| Accession | CSEP number | FWD Sequence (5’-3’) | REV sequence (5’-3’) | Amplicon length | Efficiency |
| --- | --- | --- | --- | --- | --- |
| CCU83233 | CSEP0064 | GAAACGTTCGAGCTGCAGTA | TACAGCTCCTCCTTGCCAGT | 149 | 1.24 |
| CCU82938 | CSEP0065 | GCTGCAGGGTTTTATCATGG | TCCAGACCAGCTTTCATTGG | 114 | 1.23 |
| CCU82934 | CSEP0066 | TGCCTTTAGTTGCTCACCAG | TTTCCCGGAAGCTGTTATTG | 115 | 1.23 |
| CCU83219 | CSEP0264 | CGAGATGCAGCAGTATTTGC | TGCTCTCCTTGCCAGTTTTC | 149 | 1.23 |
| CCU76783 | Conidia specific gene | GGGTTCATCGGGTCTTTTCT | AGTTGGGCCAAGGGTAAAGT | 149 | 1.22 |

**Supporting Table 4** Primers used for *H. vulgare* gene qPCR.

| Accession | gene | FWD Sequence (5’-3’) | REV sequence (5’-3’) | Amplicon length | Efficiency |
| --- | --- | --- | --- | --- | --- |
| KP293847 | GST | TGCCAGGAATTACAAGGGTTT | GGTTATTATGCTCCAGTGAAGG | 128 | 1.22 |
| KP293850 | PR5 | CGCCGACCAACTACTCAATG | GGCAGGGCAGGTGAAGG | 95 | 1.25 |
| KP293851 | PR10 | GCCAGGGTGTTCAAGACAG | CGTCCAGCCTCTCGTACTC | 142 | 1.22 |
| KP293852 | eEF1G | GGCTGCTCCTGCTAAACC | AGGGGATTCTTGGGCTTAGG | 117 | 1.23 |
| KP293845 | eEF1A | GACAGGCGATCAGGTAAGGA | TGGGCTTGGTGGGAATCAT | 91 | 1.23 |

**Supporting Table 5:** Analyses of RNA samples used for qPCR. The term ‘RIN’ stands for RNA Integrity Number; ‘hpi’ for hours post inoculation; ‘epiphytic’ for *B. graminis* epiphytic material; ‘and epidermal’ for barley epidermal peels (containing *B. graminis* hyphae).

| Time point | Replicate | Concentration(ng/ul) | RIN |
| --- | --- | --- | --- |
| 0hpi(conidia) | 1 | 126.3 | 8.5 |
|  | 2 | 133.1 | 8.3 |
|  | 3 | 209.5 | 9 |
| 0hpi(barley) | 1 | 456.4 | 9.2 |
|  | 2 | 400.8 | 8.4 |
|  | 3 | 480.0 | 8.8 |
| 4hpi epiphytic | 1 | 27.1 | 7.0 |
|  | 2 | 73.5 | 8.4 |
|  | 3 | 38.5 | 7.9 |
| 6hpi epiphytic | 1 | 219.0 | 8.6 |
|  | 2 | 75.3 | 7.0 |
|  | 3 | 79.7 | 8.2 |
| 16hpi epiphytic | 1 | 39.0 | 8.0 |
|  | 2 | 30.8 | 7.9 |
|  | 3 | 24.9 | 7.0 |
| 24hpi epiphytic | 1 | 12.3 | 8.0 |
|  | 2 | 36.7 | 6.9 |
|  | 3 | 74.9 | 8.7 |
| 24hpi epidermal | 1 | 116.0 | 9.4 |
|  | 2 | 220.0 | 9.0 |
|  | 3 | 161.5 | 9.5 |
| 48hpi epiphytic | 1 | 132.8 | 7.0 |
|  | 2 | 66.6 | 7.5 |
|  | 3 | 38.9 | 6.9 |
| 48hpi epidermal | 1 | 210.0 | 8.3 |
|  | 2 | 200.0 | 7.9 |
|  | 3 | 120.9 | 9.4 |
| 72hpi epiphytic | 1 | 369.8 | 8.7 |
|  | 2 | 270.4 | 8.0 |
|  | 3 | 155.0 | 9.2 |
| 72hpi epidermal | 1 | 160.3 | 9.0 |
|  | 2 | 160.0 | 8.0 |
|  | 3 | 113.0 | 7.9 |
| 120hpi epiphytic | 1 | 524.0 | 9.2 |
|  | 2 | 230.5 | 8.6 |
|  | 3 | 128.2 | 8 |
